# Effects of pannus formation on the flow around a bileaflet mechanical heart valve

**DOI:** 10.1371/journal.pone.0234341

**Published:** 2020-06-12

**Authors:** Woojin Kim, Haecheon Choi, Jihoon Kweon, Dong Hyun Yang, Young-Hak Kim

**Affiliations:** 1 Department of Mechanical Engineering, Seoul National University, Seoul, Korea; 2 Institute of Advanced Machines and Design, Seoul National University, Seoul, Korea; 3 Department of Cardiology, University of Ulsan College of Medicine, Asan Medical Center, Seoul, Korea; 4 Department of Radiology, University of Ulsan College of Medicine, Asan Medical Center, Seoul, Korea; Texas A&M University System, UNITED STATES

## Abstract

Some patients with a bileaflet mechanical heart valve (BMHV) show significant increases in the transvalvular pressure drop and abnormal leaflet motion due to a pannus (an abnormal fibrovascular tissue) formed on the ventricular side, even in the absence of physical contact between the pannus and leaflets. We investigate the effects of the pannus shape (circular or semi-circular ring), implantation location and height on the leaflet motion, flow structure and transvalvular pressure drop using numerical simulations. The valve model considered resembles a 25 mm masters HP valve. The mean systolic pressure drop is significantly increased with increasing pannus height, irrespective of its implantation orientation. Near the peak inflow rate, the flow behind the pannus becomes highly turbulent, and the transvalvular pressure drop is markedly increased by the pannus. At the end of valve opening and the start of valve closing, oscillatory motions of the leaflets occur due to periodic shedding of vortex rings behind the pannus, and their amplitudes become large with increasing pannus height. When the pannus shape is asymmetric (e.g., a semi-circular ring) and its height reaches about 0.1*D* (*D* (= 25 mm) is the diameter of an aorta), abnormal leaflet motions occur: two leaflets move asymmetrically, and valve closing is delayed in time or incomplete, which increases the regurgitation volume. The peak energy loss coefficients due to panni are obtained from simulation data and compared with those predicted by a one-dimensional model. The comparison indicates that the one-dimensional model is applicable for the BMHV with and without pannus.

## Introduction

A natural aortic heart valve plays an important role in the cardiovascular circulation system: it prevents oxygenated blood from flowing in the retrograde direction during the diastole phase and minimizes an interference to the blood flow during the systole phase. When a natural aortic valve does not perform its role properly due to severe diseases, it is replaced with a prosthetic heart valve. More than 50 prosthetic heart valves have been designed from its first successfully implantation by Dr. Charles Hufnagel [1, 2]. Among them, a bileaflet mechanical heart valve (BMHV) has been implanted worldwide because it shows long-lasting durability and has a lower pressure drop than caged-ball and tilting disc mechanical heart valves [2].

**Fig 1:**
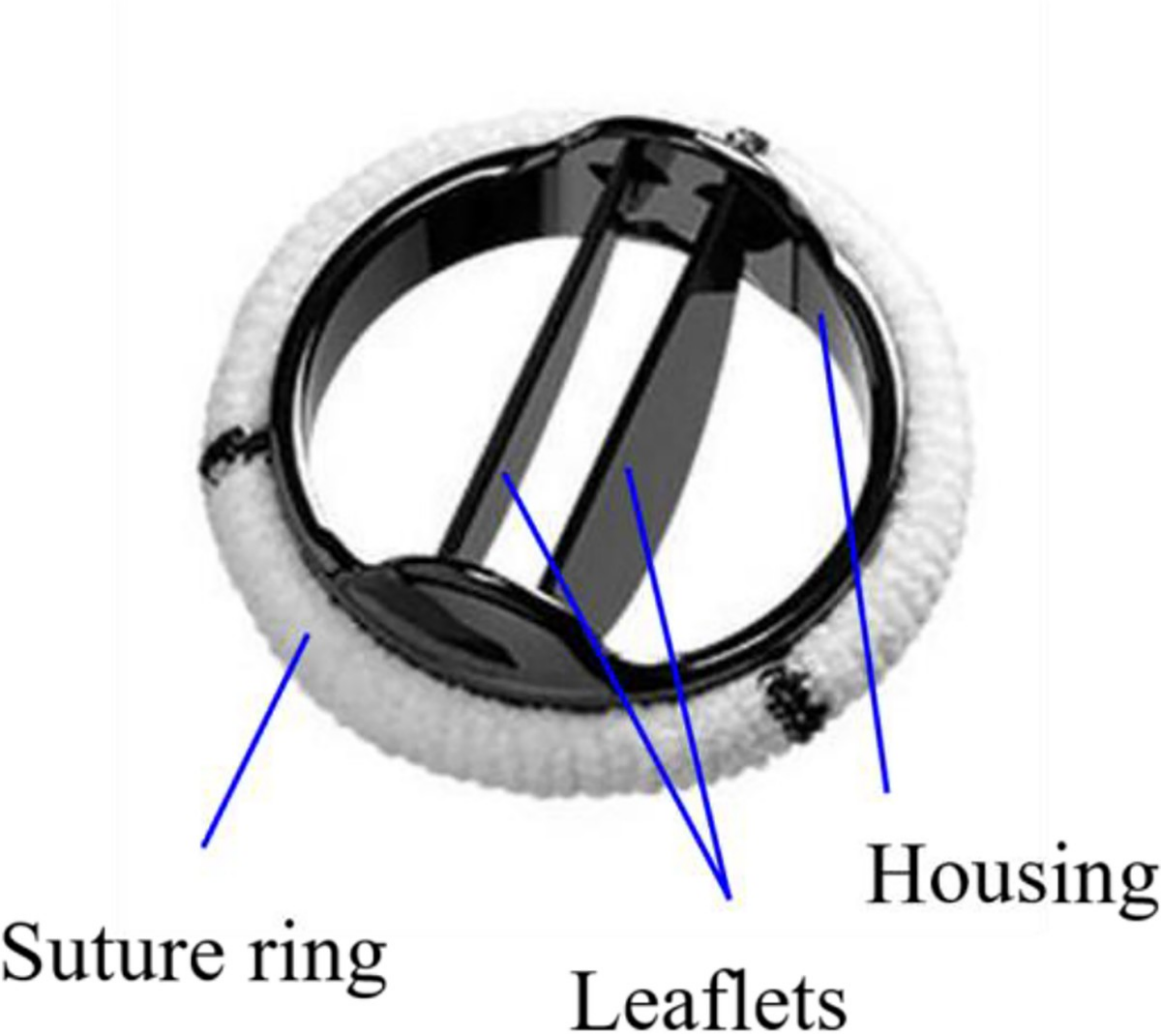
Bileaflet mechanical heart valve (BMHV): St. Jude Masters HP series valve.

A BMHV is composed of two semi-circular leaflets, housing and suture ring (Fig 1). The blood from the left ventricle runs through the central and two lateral orifices divided by the leaflets during the systole phase [3], and it flows reversely through the gaps between the leaflets and between the leaflet and housing during the diastole phase, respectively [4, 5]. Three highly turbulent jets are developed by a BMHV instead of a single jet from a natural aortic valve during the systole phase, and the regurgitant volume is higher for BMHVs than for bio-prosthetic valves [2]. These non-physiological flow patterns around a BMHV are caused by its leaflet geometry [2, 6], implantation orientation [7–11], tilt angle [7, 12], sinus of Valsalva morphology [13, 14], Valsalva graft [15], ascending aorta geometry [16], and passive control devices attached on leaflet surfaces [4]. These studies suggested better implantations of a BMHV based on the hemodynamic characteristics such as the pressure drop across a BMHV, regurgitant flow, and damage of red blood cells and plates. Ten years after the BMHV implantation, the rate of valve dysfunction was reported to range from 10% to 30% [17]. Complications such as the thrombus and pannus formation were indicated as the cause of high pressure drop across a BMHV or abnormal behaviors of valve leaflets [18, 19]. The thrombus formation on a BMHV which hampers the range of leaflet motion reduces the systolic flow area and induces significant regurgitation in the diastolic phase [17, 20]. Bouabdallaoui et al. [21] observed incomplete valve opening and closing of one leaflet induced by thrombus and a significant increase in the mean pressure drop across thrombus and a BMHV. Smadi et al. [22] showed from in vitro experiments that the maximum transvalvular pressure drop for the case of 100% malfunction (with one of two leaflets closed during a whole period) is 2.12 times that for the normal state. Adegbite et al. [23] observed high peak turbulent diffusivity for a dysfunctional valve (partially opened) from in vitro experiments.

A pannus, an abnormal layer of fibrovascular tissue growing from the suture ring to the center of a BMHV on the left ventricular side, has been proposed as a source of high transvalvular pressure drop [24–35]. Also, incomplete valve opening and closing of the leaflets have been observed in patients with pannus [25–28, 30, 36–38]. These high pressure drop and regurgitant flow are very dangerous because they result in irreversible damages to the muscle of the left ventricle [39]. It was reported that incomplete valve opening and intermittent incomplete valve closing of one leaflet occur in patients with pannus even if there is no physical contact between the leaflets and pannus [28, 31]. This indicates that the flow disturbances caused by pannus should be one of the causes of incomplete leaflet motions. Ha et al. [40] modelled symmetric and asymmetric panni as circular and semi-circular rings, respectively, and found abnormal behaviors of valve leaflets through in vitro experiments: incomplete valve opening and complete valve closing for the symmetric pannus with the ratio of its height to the diameter of an aorta greater than 0.1. However, the peak bulk velocity used in the in vitro experiments [40] is much lower than that obtained from healthy individuals [2, 41] and other in vitro experiments for BMHV [3, 42].

In this study, we explain the mechanism of incomplete opening and closing of a BMHV based on the hemodynamic interplay between the pannus and BMHV under a physiological inflow, even if there is no physical contact between the pannus and leaflets. We also investigate the transvalvular pressure drop and flow characteristics caused by pannus formation, as compared to those by thrombus.

## Methods

**Fig 2:**
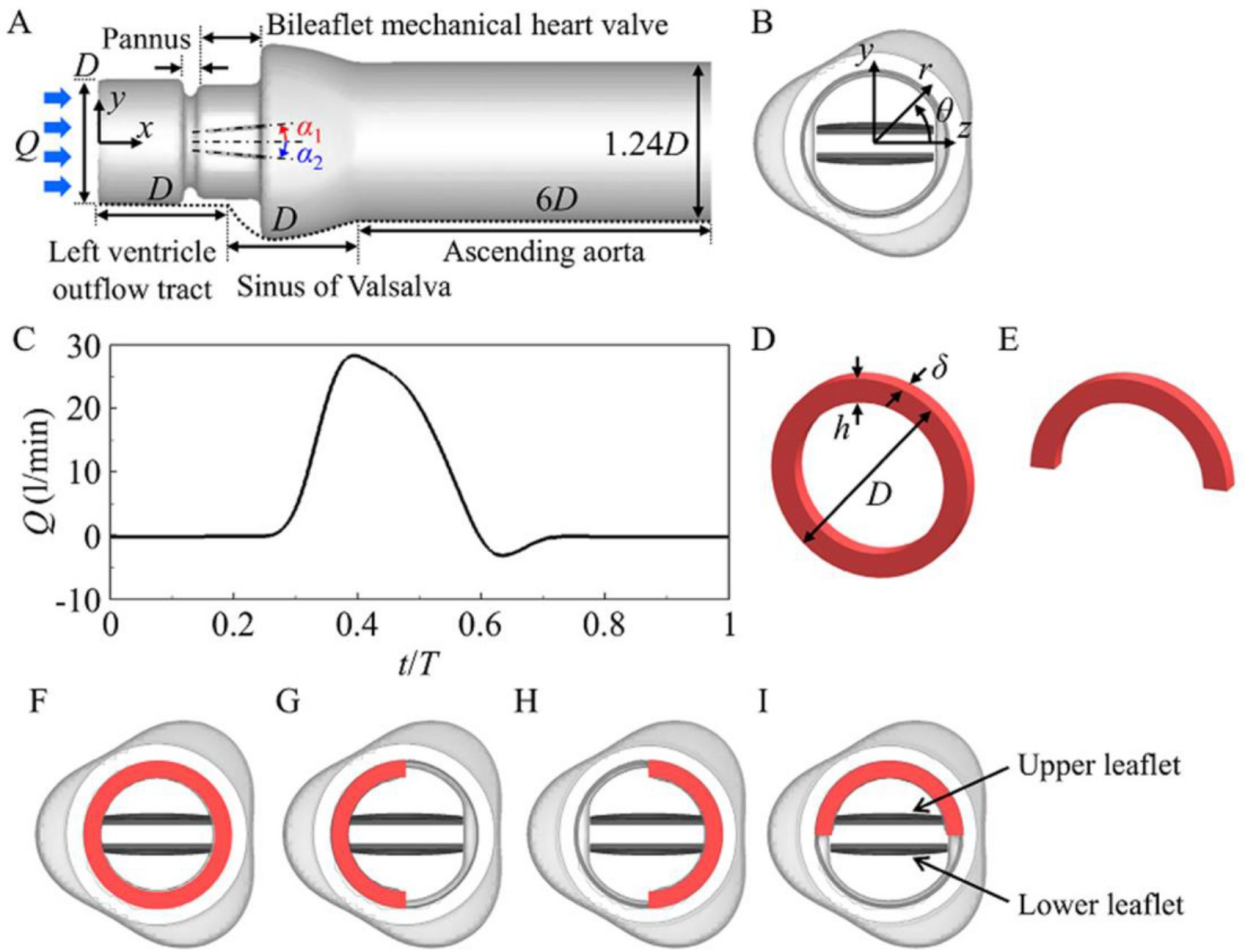
Computational setup. (A) Schematic diagram of an aorta model. (B) Front view of the aorta model without pannus. (C) Temporal variation of inflow rate (*Q*) during a cardiac cycle (*T*). (D) Circular and (E) semi-circular types of pannus formation. Front views of the aorta models with (F) circular pannus, (G) left semi-circular pannus, (H) right semi-circular pannus, and (I) upper semi-circular pannus. *D*, *α*_1_ and *α*_2_ in (A) are the diameter of aorta inlet and angles of upper and lower leaflets, respectively. The pannus heights (*h*) considered in (D) and (E) are 0.07*D* to 0.14*D*, and the pannus thickness (*δ*) is fixed at 0.1*D*. The panni in (D)-(I) are colored in red.

The schematic diagram of an aorta model is shown in Fig 2A. Left ventricle outflow tract (LVOT) and ascending aorta are modelled as a straight pipe with the diameters of *D* (= 25 mm) and 1.24*D*, respectively. The cross-section of sinus of Valsalva is described as an epitrochoid with three bulges [14, 43]. The lengths of LVOT, sinus of Valsalva and ascending aorta are *D*, *D* and 6*D*, respectively. The valve model considered resembles a 25 mm maters HP valve (St. Jude Medical Inc., St. Paul, MN) and is placed in a supra-annular position. The leaflet orientation is planar symmetric with respect to sinus of Valsalva (Fig 2B). The opening and closing angles of the leaflets with respect to the streamwise direction (*x*) are *α*_*open*_ = 5° and *α*_*close*_ = 65°, respectively [44]. Blood is assumed as a Newtonian fluid with the density (*ρ*_*b*_) of 1060 kg/m^3^ and dynamic viscosity (*μ*_*b*_) of 3.7×10^3^ Pa∙s, because leaflet motion and pressure drop show little differences between Newtonian and non-Newtonian fluids [45]. Physiological inflow rate is shown in Fig 2C [42], where the peak inflow rate (*Q*_*peak*_) is 28 liter/min, the mean inflow rate (*Q*_*mean*_) is 5 liter/min, and the period of a cardiac cycle (*T*) is 866 ms. The peak Reynolds number (Re = *ρ*_*b*_*U*_*p*,*bulk*_*D*/*μ*_*b*_) is 6872, where *U*_*p*,*bulk*_ (= *Q*_*peak*_/(0.25π*D*^2^)) is the peak bulk velocity in LVOT. The density (*ρ*_*l*_) and moment of inertia of the leaflets are 2000 kg/m^3^ and 6.52×10^−9^ kg∙m^2^, respectively.

A pannus has been observed in a patient implanted with a masters HP valve [46]. It is modelled as a circular ring (Fig 2D) or a semi-circular ring (Fig 2E) with a thickness of *δ* = 0.1*D*. The pannus is situated on the left ventricle side of a BMHV, and the distance between the pannus and a BMHV is 0.05*D*, which allows no physical contact between them. The effect of the pannus height is examined by varying *h* from 0.07*D* (slightly higher than the housing height (0.05*D*) of a BMHV) to 0.14*D* (*h* = 3.5 mm observed in a patient [27]). The effects of the pannus shape and growth orientation are also investigated with a fixed pannus height of *h* = 0.12*D* (*h* = 3 mm): a circular pannus and three cases of different growth orientation for a semi-circular pannus (left, right and upper arrangements) (Fig 2F–2I). To compare the pressure drop induced by the pannus formation with that induced by a thrombus, three cases of restricted opening angles (*α*_*open*_ = 10°, 15°, and 20°) are also considered. Note that patients with a thrombus showed *α*_*open*_ = 17.5° ~ 53° despite normal valve closing [20].

The governing equations of unsteady incompressible viscous fluid flow for large eddy simulation (LES) are the filtered Navier-Stokes and continuity equations. A cylindrical coordinate system is introduced and a discrete-forcing immersed boundary (IB) method [47] is used to satisfy the no-slip condition on the leaflet surfaces and aorta wall. This immersed boundary method enables a sharp representation of an interface so it is desirable for high Reynolds number flows [48]. The non-dimensional forms of the governing equations are

∂u˜i∂t+∂u˜iu˜j∂xj=−∂p˜∂xi+1Re∂2u˜i∂xj∂xj−∂τij∂xj+fi, (1)

$$\frac{\partial {\tilde{u}}_{i}}{\partial x_{i}}-q=0,$$ (2)

where *t* is the time, *x*_*i*_ are the coordinates, ${\tilde{u}}_{i}$ are the corresponding filtered velocity components, $\tilde{p}$ is the filtered pressure, $\tau_{ij}=\tilde{u_{i}u_{j}}-{\tilde{u}}_{i}{\tilde{u}}_{j}$ is the subgrid-scale (SGS) stress tensor, and $\tilde{\left(\cdot \right)}$ is the filtering operation for LES. *f*_*i*_ and *q* are the momentum forcing and mass source/sink, respectively, for satisfying the no-slip condition on the immersed boundary (the leaflet surfaces and aorta wall) and the continuity for the cells containing the immersed boundary (see [47] for the detail). A dynamic global eddy viscosity model [49, 50] is used to determine the SGS stress tensor *τ*_*ij*_. All variables are non-dimensionalized by the peak bulk velocity in LVOT (*U*_*p*,*bulk*_), the diameter of an aorta (*D*), and the blood density (*ρ*_*b*_). For time advancement, a second-order semi-implicit fractional step method [51] is used to solve Eqs (1) and (2): the Crank-Nicolson method is applied to spatial derivative terms in the azimuthal direction within the core region (0 ≤ *r*/*D* < 0.2) and those in the radial direction within the outer region (*r*/*D* ≥ 0.2), and a third-order Runge-Kutta method is used for other terms. The second-order central difference scheme is applied to all the spatial derivative terms.

The rotational motions of the leaflets are described by

$$\frac{I_{l}}{\rho_{b}D^{5}}\frac{d^{2}\alpha_{i}}{dt^{2}}=M_{i}\;(i=1:\;\mathrm{upper}\;\mathrm{leaflet},\;2:\;\mathrm{lower}\;\mathrm{leaflet}),$$ (3)

where *α*_*i*_ is the angle of each leaflet (Fig 2A) and *M*_*i*_ is the rotational-axis component of the torque on each leaflet by fluid flow. Here, *M*_*i*_ is positive when the torque on each leaflet imposes in the direction from the opening angle to the closing angle. A second-order implicit generalized-alpha method [52] is applied to solve Eq (3). Since the governing equations of fluid flow and leaflet motion are semi-implicitly and implicitly solved, respectively, an iterative method is applied to satisfy the no-slip condition and impose the moment on the surfaces of the leaflets: during one iteration, Eqs (1) and (2) are first solved by satisfying the no-slip condition on the leaflet surfaces obtained at the previous iteration step, then Eq (3) is solved with the torques imposed on the surfaces obtained at the previous iteration step, and then iterations go on until $\left|d^{2}\alpha_{i}^{k}/dt^{2}-d^{2}\alpha_{i}^{k-1}/dt^{2}\right|\leq 10^{-4}$, where *k* is the iteration index. Since the density ratio of the leaflet to fluid (≈ 1.887) is low, an under-relaxation method [42, 53] is applied to the angles, angular velocities and angular accelerations of the leaflets for stable solutions, together with an Aitken’s acceleration method [54] for reduction of computational cost.

**Fig 3:**
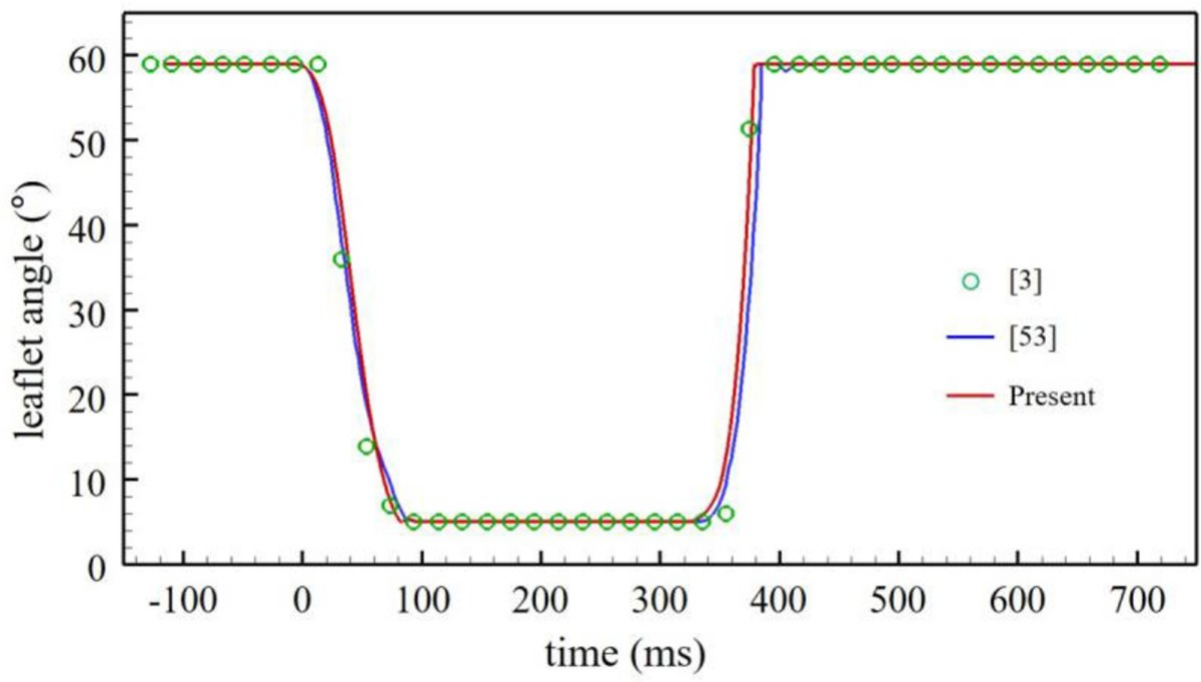
Time history of the leaflet angle during one cycle obtained from the present study, in comparison with those from previous numerical [53] and experimental [3] studies.

The numbers of grid points used in the streamwise, radial, and azimuthal directions are [*N*_*x*_, *N*_*r*_, *N*_*θ*_] = [305, 185, 300] for the aorta model without pannus, and are increased by about two to three times depending on the types of the pannus formation. For example, in case of Fig 2F, [*N*_*x*_, *N*_*r*_, *N*_*θ*_] = [417, 287, 400]. The grid resolutions are obtained from an extensive grid independence study by increasing the number of grid points by 1.5 times in each direction, resulting in less than 3% changes in the peak plane-averaged net pressure drop. The size of computational time step is determined by the CFL (Courant-Friedrichs-Lewy) number ≤ 1.5 [51]; for example, Δ*t*/*T* = 2.92×10^−5^ ~ 9.72×10^−5^ for the aorta model without pannus, and 1.33×10^−5^ ~ 4.18×10^−5^ for the aorta model with the circular pannus of *h* = 0.14*D*. The (inlet) length of LVOT is *D*, and the inlet streamwise velocity is given as *ũ*_*x*_(*t*, *r*) = *u*_*c*_(*t*)tanh[60(1-2*r*/*D*)] for 0 ≤ *r*/*D* ≤ 0.5, where *u*_*c*_(*t*) = 1.303*Q*(*t*)/*D*^2^ (Fig 2C), and other velocity components at the inlet are zero, following the inlet length and velocity profile of Tullio et al. [42]. At the outlet, the Neumann boundary conditions, ∂*ũ*_*x*_/∂*x* = ∂*ũ*_*r*_/∂*x* = ∂*ũ*_*θ*_/∂*x* = 0, are imposed, where *ũ*_*r*_ and *ũ*_*θ*_ are the filtered radial and azimuthal velocity components. We test the present numerical method for a flow through a BMHV (similar to the present flow problem) considered in experimental [3] and numerical [53] studies. As shown in Fig 3, the leaflet angle variation in time obtained from the present method is in an excellent agreement with those from the previous studies [3, 53].

The minimal cross-sectional area of a BMHV is defined as a geometric orifice area (*GOA*) [55], and the present *GOA* (*GOA* = 3.59 cm^2^) is close to that specified by manufacturer (*GOA* = 3.67 cm^2^). The ensemble-averaged peak effective orifice area (*EOA*_*p*,*Dop*_)^*ens*^ of the prosthesis is calculated with the following continuity equation by mean of the simplified peak velocity method [56]:

$${\left(EOA_{p,Dop}\right)}^{ens}=CSA\left[{\left(u^{ens}\right)}_{x,LVOT,peak}/{\left(u^{ens}\right)}_{x,valve,peak}\right],$$ (4)

where *CSA* is the cross-section area of LVOT, (*u*^*ens*^*)*_*x*,*LVOT*,*peak*_ and (*u*^*ens*^)_*x*,*valve*,*peak*_ are the maximum ensemble-averaged streamwise velocities at the peak inflow rate in LVOT and right after a BMHV, respectively, and ^*ens*^ indicates the ensemble averaging. Hereafter, the number of cycles used for the ensemble averaging is equal to or greater than seven because the differences between (*EOA*_*p*,*Dop*_)^*ens*^ obtained from seven and ten cycles are less than 2.2% for no, upper and right semi-circular panni with *h* = 0.12*D*. For the flow around a BMHV without pannus, the present (*EOA*_*p*,*Dop*_)^*ens*^ of 2.78 cm^2^ agrees well with the product specification provided by the manufacturer ((*EOA*_*p*,*Dop*_)^*ens*^ = 3.08 cm^2^).

## Results

### Motions of the leaflets

**Fig 4:**
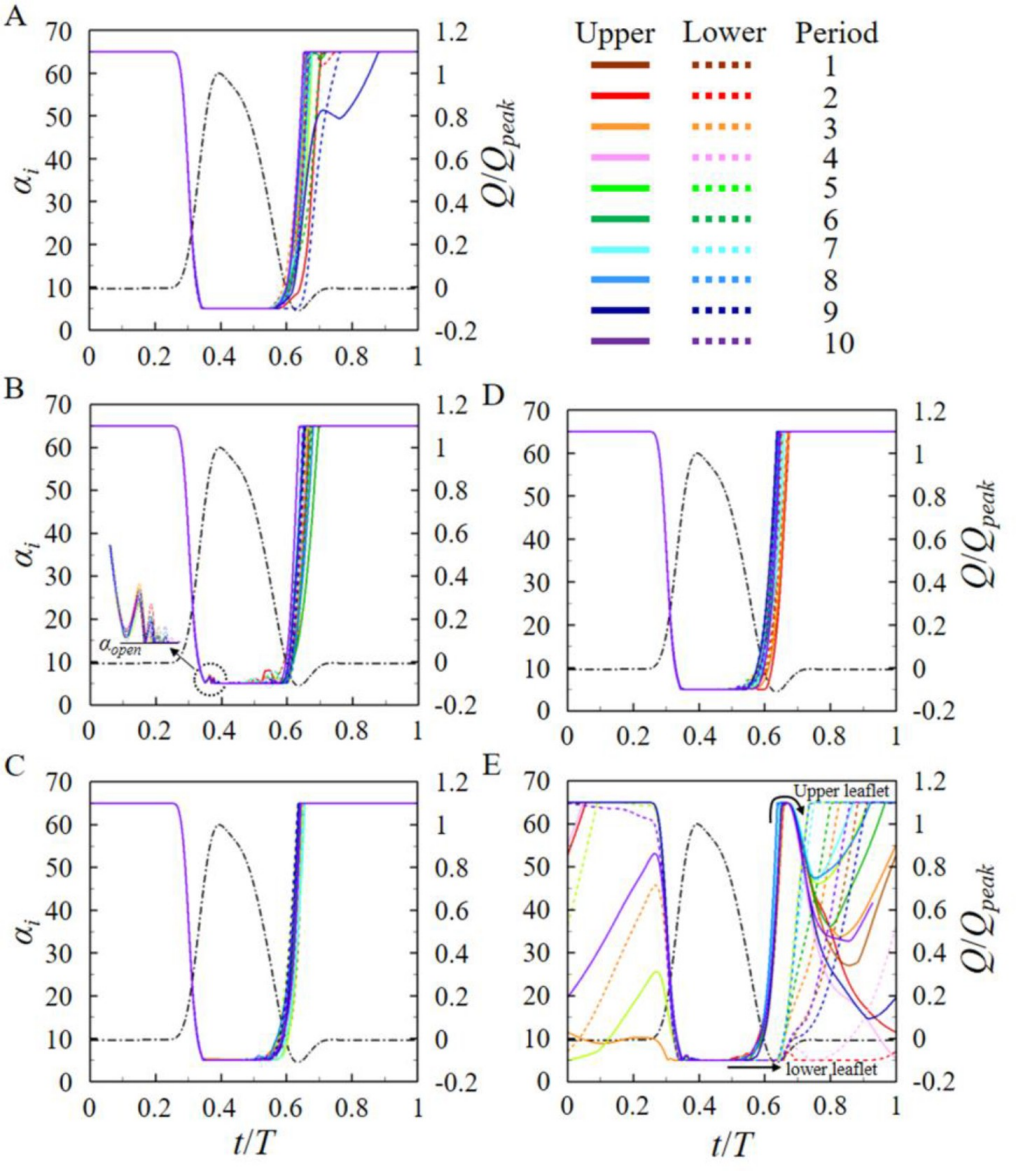
Time histories of the angles of the upper and lower leaflets during ten cycles. (A) No pannus. (B) Circular pannus. (C) Left semi-circular pannus. (D) Right semi-circular pannus. (E) Upper semi-circular pannus. Here, the black chain-dotted line in each figure is the inflow rate normalized by the peak inflow rate, and the colored solid and dashed lines denote the motions of upper and lower leaflets during ten cycles, respectively. The height of pannus is *h* = 0.12*D*.

Fig 4 shows the temporal variations of the leaflet angles for different pannus formations, together with those of no pannus. Here, the leaflets are fully open at *α* = 5° and closed at *α* = 65°. For the four cases considered (no, circular, left semi-circular, and right semi-circular panni; Fig 4A–4D), the leaflets start to open (from *α* = 65°) at the same time (at *t*/*T* = 0.26) during ten cycles and reach *α* = 5° (full opening) after time duration of about 0.09*T*, although the circular pannus causes weak oscillatory motions of the leaflets near the end of valve opening and slightly delays its completion. As shown in Fig 4A, an irregular motion of the leaflet sometimes occurs during the closing phase (i.e., α = 5° to 65°). However, in the case of the upper semi-circular pannus (Fig 4E), the motion of the leaflets is very irregular during both the opening and closing phases. The leaflets are not completely closed and even remain open for three out of ten cycles. This incomplete closing of the leaflets seriously deteriorates the performance of a BMHV (see below). These irregular and non-periodic motions of the leaflets are prominent especially during the valve closing phase, and the reason responsible for these motions is explained in terms of flow interaction between the pannus and leaflets in the following section.

### Flow interaction with the leaflets

**Fig 5:**
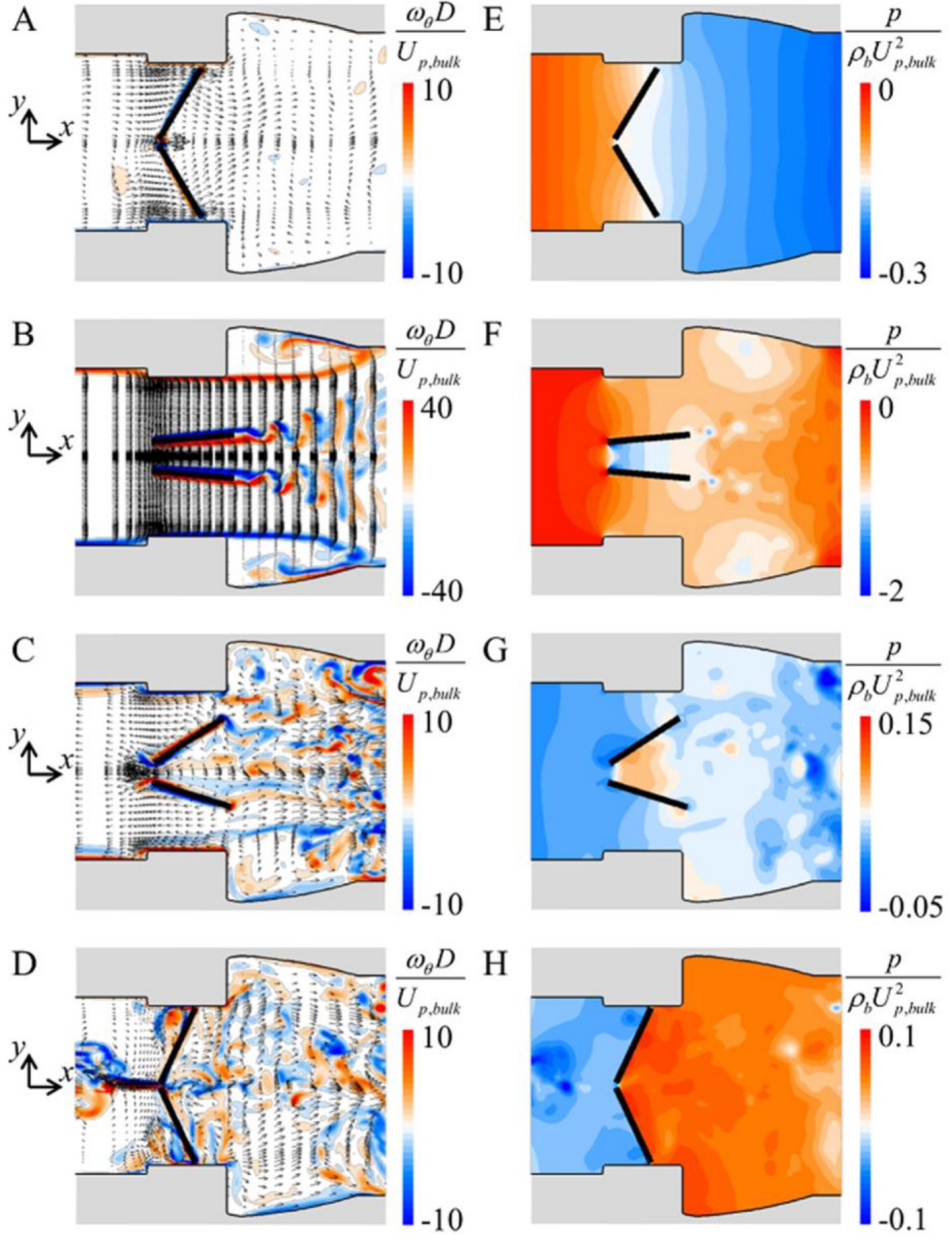
No pannus: contours of the instantaneous azimuthal vorticity and velocity vectors (A-D) and contours of the instantaneous pressure (E-H) during 10^th^ cycle (A and E: *t*/*T* = 9.28; B and F: *t*/*T* = 9.39; C and G: *t*/*T* = 9.63; D and H: *t*/*T* = 9.70) on the (*x*-*y*) plane (*θ* = 90°). Here, different contour levels are selected to better represent the flow features.

Fig 5 shows the contours of the instantaneous azimuthal vorticity and pressure and instantaneous velocity vectors during 10^th^ cycle for the aorta model without pannus. While the inflow rate increases from zero to the peak value (9.26 ≤ *t*/*T* ≤ 9.39; Fig 2C), three jets develop from the left ventricle to the ascending aorta (Fig 5B) and the valve opens by favourable pressure gradients formed in this region (Fig 5E and 5F). In the sinus of Valsalva, shear layers develop from the housing and vortex shedding occurs from the trailing edges of the leaflets (Fig 5B). While the inflow rate decreases from zero to the negative peak value (9.60 ≤ *t*/*T* ≤ 9.63; Fig 2C), a reverse flow occurs from the inclined leaflets to the left ventricle (Fig 5C), and the valve is closed by an adverse pressure gradient formed from the ascending aorta to the left ventricle (Fig 5G). After reaching the closing angle (*t*/*T* ≥ 9.67), the valve closing is maintained for about 0.33*T* during 10^th^ cycle because of the pressure difference acting on the leaflet surfaces (Fig 5H).

**Fig 6:**
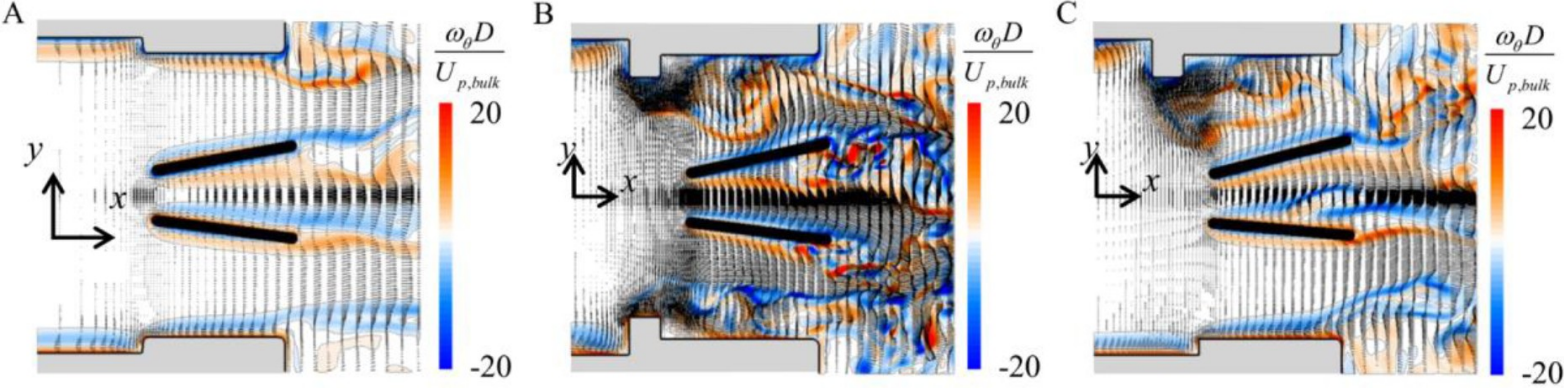
Contours of the instantaneous azimuthal vorticity and instantaneous velocity vectors on an (*x*-*y*) plane at *t*/*T* = 9.60 (*h* = 0.12*D*). (A) no pannus. (B) Circular pannus. (C) Upper semi-circular pannus.

**Fig 7:**
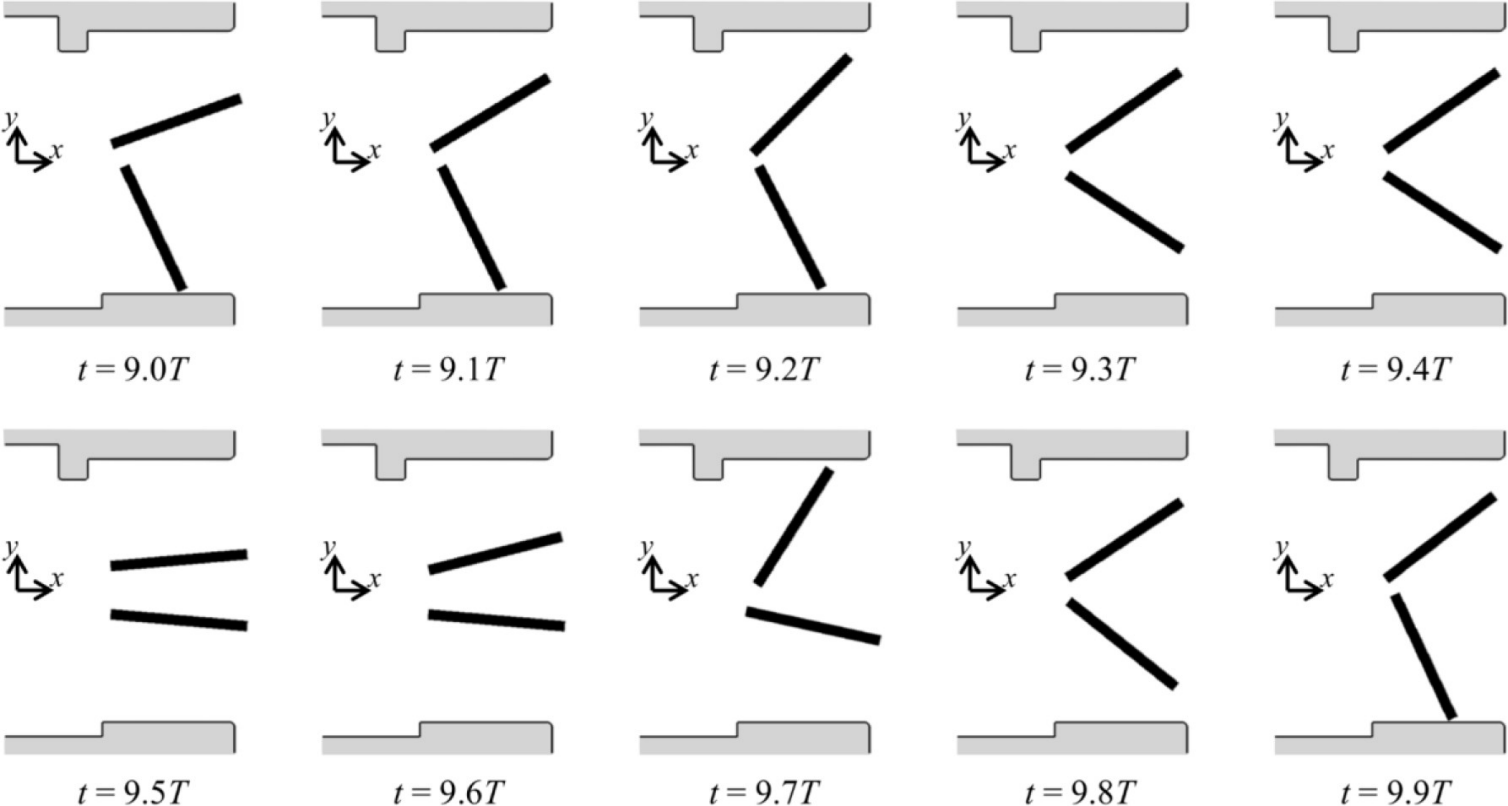
Leaflet motion during a cardiac cycle (*T*) for the case of upper semi-circular pannus (*h* = 0.12*D*).

Fig 6 shows the contours of the instantaneous azimuthal vorticity and velocity vectors at *t*/*T* = 9.60 (at an initial valve-closing period) for the cases of no pannus, circular pannus and upper semi-circular pannus. Without pannus, the flow field in between the housing and leaflet is relatively calm except the shear layer evolution behind the leaflets (Fig 6A). The presence of pannus strongly changes the flow field in between the housing and leaflet. Flow separation and vortex evolution are observed right behind the pannus (Fig 6B and 6C). Especially, the flow fields in the upper and lower parts are very different for the case of upper semi-circular pannus, which causes asymmetric motions of upper and lower leaflets and finally imperfect closing of the leaflets (Fig 7).

**Fig 8:**
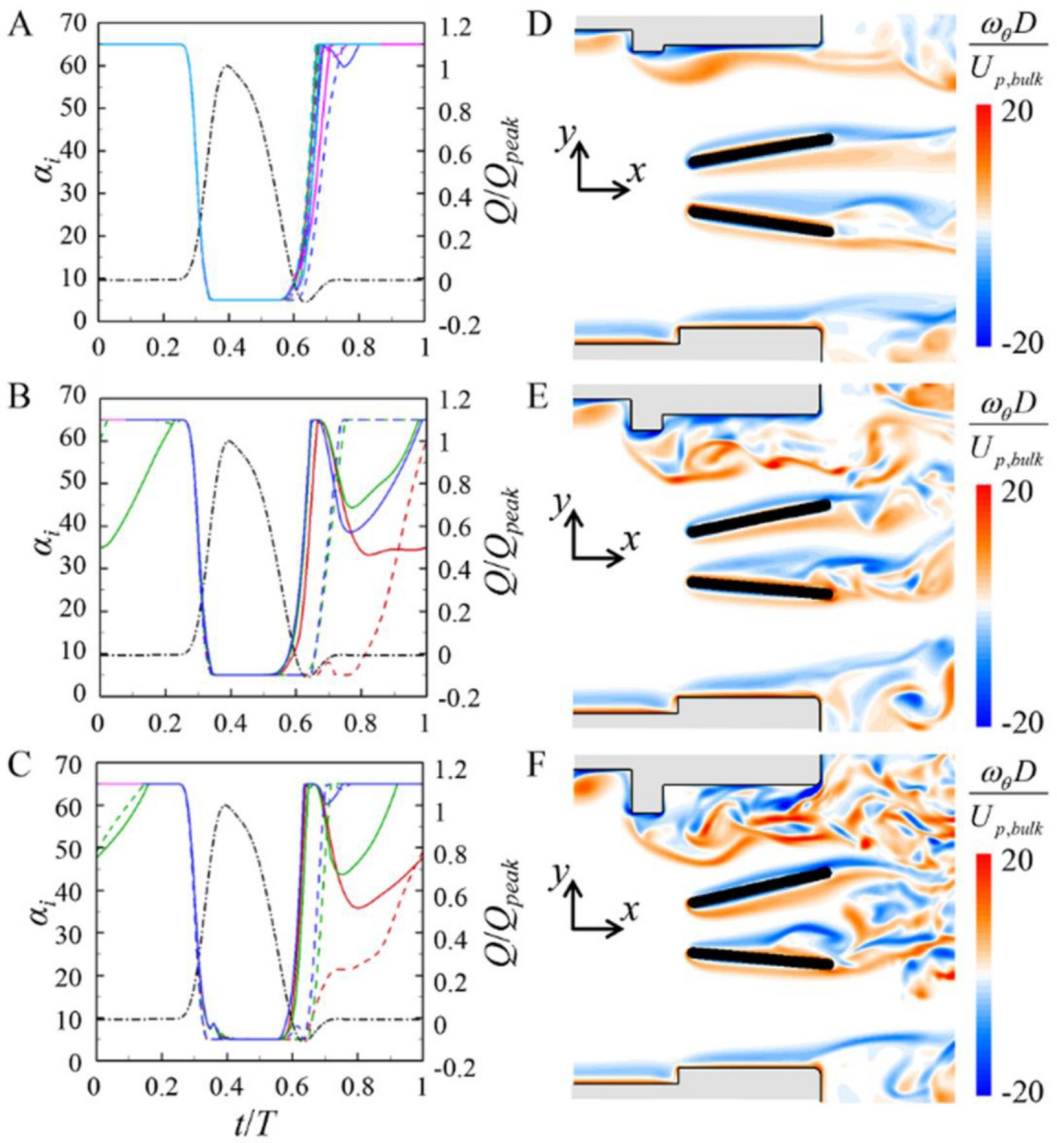
Effect of the pannus height (*h*) on the upper and lower leaflet motions and flow fields for the case of upper semi-circular pannus. (A)–(C) Time histories of the upper (solid line) and lower (dashed line) leaflet angles. (D)–(F) Contours of the instantaneous azimuthal vorticity (*t*/*T* = 0.60). Here, (A) and (D) are for *h* = 0.07*D*, (B) and (E) are for *h* = 0.1*D*, and (C) and (F) are for *h* = 0.14*D*, respectively. ▬▬, 0-*T*; ▬▬, *T*-2*T*; ▬▬, 2*T*-3*T*; ▬▬, 3*T*-4*T*; ▬▬, 4*T*-5*T*. The chain-dotted lines are the inflow rate normalized by the peak inflow rate.

In the case of upper semi-circular pannus, incomplete or delayed valve closing is observed for the pannus height of *h* = 0.12*D*. Thus, the effect of the height of upper semi-circular pannus (*h* = 0.07*D*, 0.10*D*, and 0.14*D*) is further investigated. Fig 8 shows the effect of the pannus height on the leaflet motions and the flow fields for the case of upper semi-circular pannus. For *h* = 0.07*D*, the leaflet motions are not very different from those of no pannus (Fig 4A). However, for *h* = 0.10*D* and 0.14*D*, the leaflet closing is significantly delayed as observed for *h* = 0.12*D*. For *h* = 0.07*D*, only weak shear layer evolves from the pannus, whereas, for *h* = 0.10*D* and 0.14*D*, the asymmetry in the vortical structures between the upper and lower parts becomes severer for higher pannus height, resulting in abnormal valve closing. The effect of the pannus height on the pressure drop is discussed below.

### Transvalvular pressure drop

**Fig 9:**
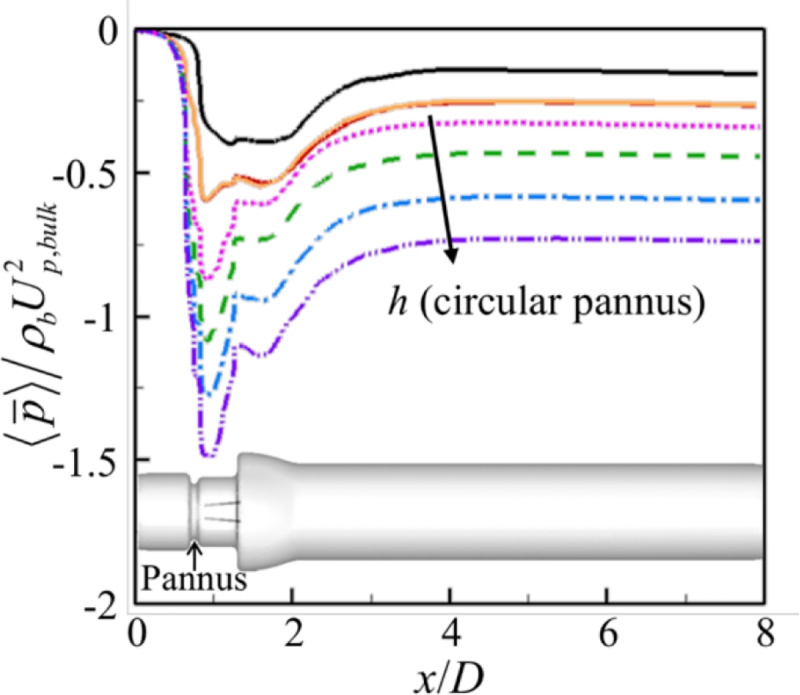
Mean systolic plane-averaged pressures along the streamwise direction for the cases with and without pannus. **▬▬**, No pannus; ▬▬, left semi-circular pannus (*h*/*D* = 0.12); ▬▬, upper semi-circular pannus (*h*/*D* = 0.12); ▪▪▪, circular pannus (*h*/*D* = 0.11); ▬▬▬, circular pannus (*h*/*D* = 0.12); ▬▪▬, circular pannus (*h*/*D* = 0.13); ▬▪▪▬, circular pannus (*h*/*D* = 0.14).

Fig 9 shows the mean systolic plane-averaged pressure along the streamwise direction. Without pannus, the pressure rapidly decreases just before the leading edges of the leaflets due to the instalment of BMHV, is nearly constant across the BMHV and sinus of Valsalva, and recovers in the ascending aorta. Once the pressure reaches maximum around *x* = 4*D*, it slowly decreases due to friction at the wall. With circular pannus, the maximum pressure drop occurs after the pannus. The amount of maximum pressure drop due to the pannus is very large and increases with increasing pannus height. Among the different panni with *h* = 0.12*D*, the maximum pressure drop occurs for the circular pannus because of its largest area blockage, whereas the difference in the pressure drops from left and upper semi-circular panni is not so large (see Discussion section).

### Effect of the restricted leaflet opening angle

**Fig 10:**
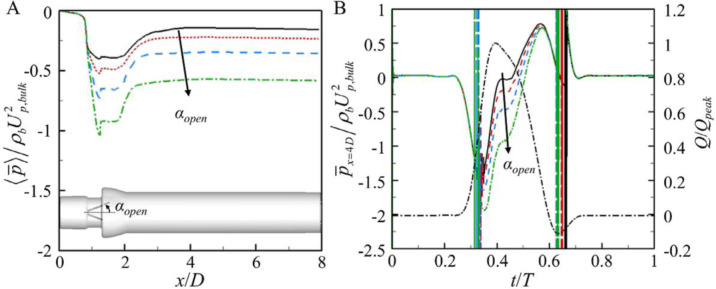
Variation of the plane-averaged pressure with the restricted opening angle (no pannus). (A) Mean systolic plane-averaged pressure along the streamwise direction. (B) Instantaneous plane-averaged pressure at *x*/*D* = 4 during one cycle. ▬▬, *α*_*open*_ = 5°; ▪▪▪▪, 10°; ▬▬▬, 15°; ▬▪▬, 20°. In (B), ▬ ▪ ▬, inflow rate normalized by the peak inflow rate.

**Fig 11:**
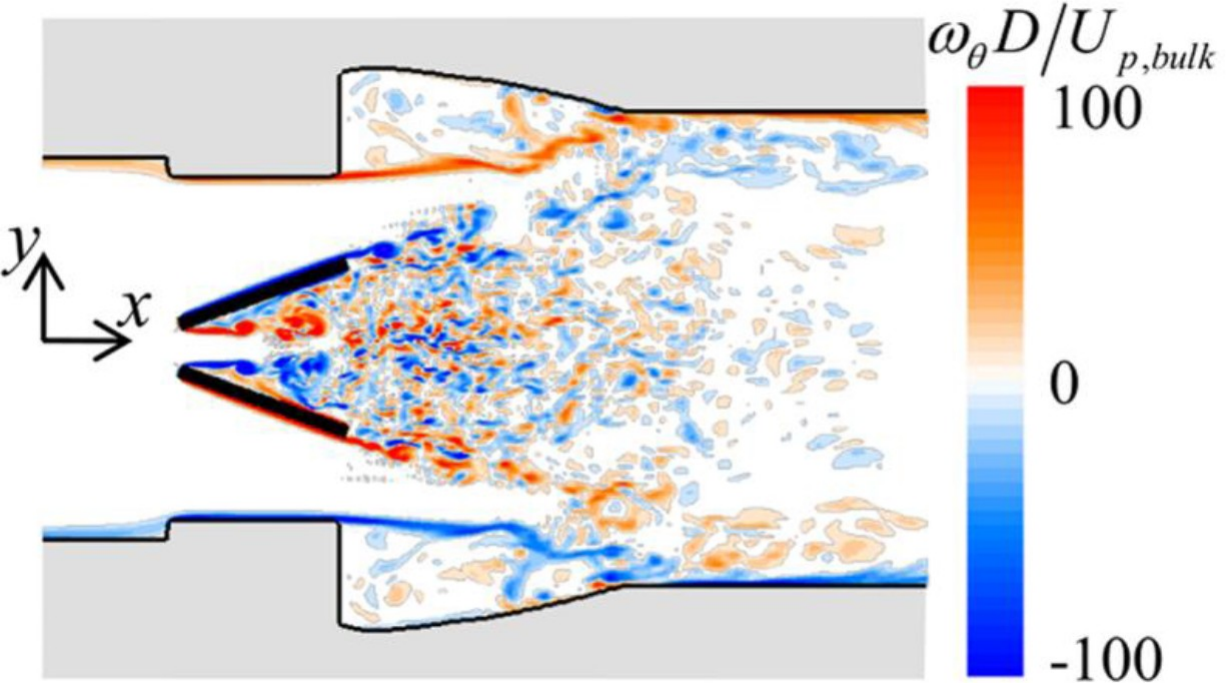
Contour of the instantaneous azimuthal vorticity (*ω*_*θ*_) at the peak inflow rate (*t*/*T* = 0.4) for *α*_*open*_ = 20°.

So far, we considered the leaflet opening angle of *α*_*open*_ = 5°. In this section, we investigate the effect of the opening angle restriction (*α*_*open*_ = 5°, 10°, 15°, and 20°) on the pressure drop by considering the situation that a thrombus restricts the opening angle of the leaflets. Fig 10A shows the variation of the mean systolic phase-averaged pressure along the streamwise direction for four different restricted leaflet opening angles. As shown, a more restricted opening angle (i.e., increasing *α*_*open*_) causes a higher pressure drop, and maximum pressure drop occurs at the tip of the leaflets. Fig 10B shows the time histories of the plane-averaged pressure at *x* = 4*D*. As shown, the increase in the mean pressure drop with increasing opening angle is mainly from large pressure drop near the peak inflow rate. The wide wake region due to a large opening angle causes a large pressure drop at the peak inflow rate (Fig 11).

## Discussion

Table 1 summarizes the mean systolic plane-averaged maximum and net pressure drops. $\langle \Delta {\bar{p}}_{\max}\rangle$ and $\langle \Delta {\bar{p}}_{net}\rangle$, respectively, peak plane-averaged net pressure drop $\Delta {\bar{p}}_{p,net}$, ensemble-averaged peak effective orifice area ${\left(EOA_{p,Dop}\right)}^{ens}$, and peak energy loss coefficient *EL_peak_* for the various cases considered in the present study. The flow area reduced by the pannus increases with increasing pannus height, which results in the increase in the maximum and net pressure drops. The maximum and net pressure drops are also affected by the pannus shape. The upper semi-circular pannus increases the pressure drops more than the right (and left) semi-circular pannus, which is expected from the flow modifications described in the previous section. The net pressure drop for the upper semi-circular pannus of *h* = 0.14*D* is slightly higher than that for the circular pannus of *h* = 0.11*D*, although the flow area reduced by the latter (0.31*D*^2^) is larger than that by the first (0.19*D*^2^). This indicates that the net pressure drop is affected by both the pannus area and shape. The opening angle restriction also causes significant net pressure drop. The net pressure drops with *α*_*open*_ = 15° and 20° are similar to those by circular panni of *h* = 0.11*D* and 0.13*D*, respectively, and it increases with increasing opening angle restriction. In many case studies [21–32], the maximum pressure drop has been used as one of diagnostic tools for determining the condition of a BMHV patient with pannus. However, it does not exactly represent the severity of pannus formation. For example, the maximum pressure drop for the case of circular pannus with *h* = 0.12*D* was slightly larger than that for the case with the restricted opening angle of *α*_*open*_ = 20°, but the net pressure drop of the first was smaller than that of the latter (see Table 1). Therefore, some other diagnostic tools have been suggested. Garcia et al. [57] applied the one-dimensional continuity and momentum conservation together with the Bernoulli equation to a pipe with an orifice, and obtained the following relation for the peak energy loss *EL*_*peak*_:

$$\frac{{\left(EOA_{p,Dop}\right)}^{ens}\times A_{A}}{A_{A}-{\left(EOA_{p,Dop}\right)}^{ens}}=\frac{Q_{peak}\sqrt{0.5\rho_{b}}}{\sqrt{EL_{peak}}}.$$ (5)

Here, (*EOA*_*p*,*Dop*_)^*ens*^ is given in Eq (4), *A*_*A*_ is the cross-section area of an ascending aorta, and *EL*_*peak*_ is defined by

$$EL_{peak}=\Delta {\bar{p}}_{p,net}+\frac{1}{2}\rho_{b}\left[{\left\{U_{p,bulk}\right\}}^{2}-{\left\{U_{A,p,bulk}\right\}}^{2}\right],$$ (6)

where *U*_*A*,*p*,*bulk*_ and $\Delta {\bar{p}}_{p,net}$ are the peak bulk velocity and peak plane-averaged net pressure drop at an ascending aorta, respectively. Using Eq (5), the peak energy loss coefficient (in a non-dimensional form) is obtained as follows:

$$C_{EL}\equiv \frac{EL_{peak}}{0.5\rho_{b}Q_{peak}^{2}/A_{A}^{2}}={\left[\frac{A_{A}}{{\left(EOA_{p,Dop}\right)}^{ens}}-1\right]}^{2}.$$ (7)

**Table 1:** Mean systolic plane-averaged maximum and net pressure drops, peak plane-averaged net pressure drop, ensemble-averaged peak effective orifice area, and peak energy loss coefficient. Here, the net pressure drop is the pressure difference between at *x*/*D* = 0 and 4, and *A*_*r*_ is the flow area reduced by pannus.

	No pannus	Circular pannus				Semi-circular pannus			Opening angle restriction		
						Upper		Right	(no pannus)		
*α*_*open*_ (°)	5	5				5		5	10	15	20
*h*/*D*	-	0.11	0.12	0.13	0.14	0.12	0.14	0.12	-	-	-
*A*_*r*_/*D*^2^	-	0.31	0.33	0.36	0.38	0.17	0.19	0.17	-	-	-
$\frac{\langle \Delta {\bar{p}}_{\max}\rangle }{\rho_{b}U_{p,bulk}^{2}}$	0.40	0.87	1.09	1.28	1.49	0.61	0.73	0.60	0.52	0.73	1.04
$\frac{\langle \Delta {\bar{p}}_{net}\rangle }{\rho_{b}U_{p,bulk}^{2}}$	0.14	0.33	0.44	0.59	0.74	0.27	0.34	0.25	0.22	0.35	0.57
$\frac{\Delta {\bar{p}}_{p,net}}{\rho_{b}U_{p,bulk}^{2}}$	0.32	0.73	0.91	1.24	1.61	0.59	0.77	0.56	0.49	0.75	1.24
$\frac{{\left(EOA_{p,Dop}\right)}^{ens}}{D^{2}}$	0.44	-	0.35	0.33	0.30	0.41	-	0.40	-	0.36	0.32
$\frac{2EL_{peak}}{\rho_{b}Q_{peak}^{2}/A_{A}^{2}}$	2.85	4.83	5.69	7.24	8.95	4.15	5.01	3.99	3.66	4.92	7.21

**Fig 12:**
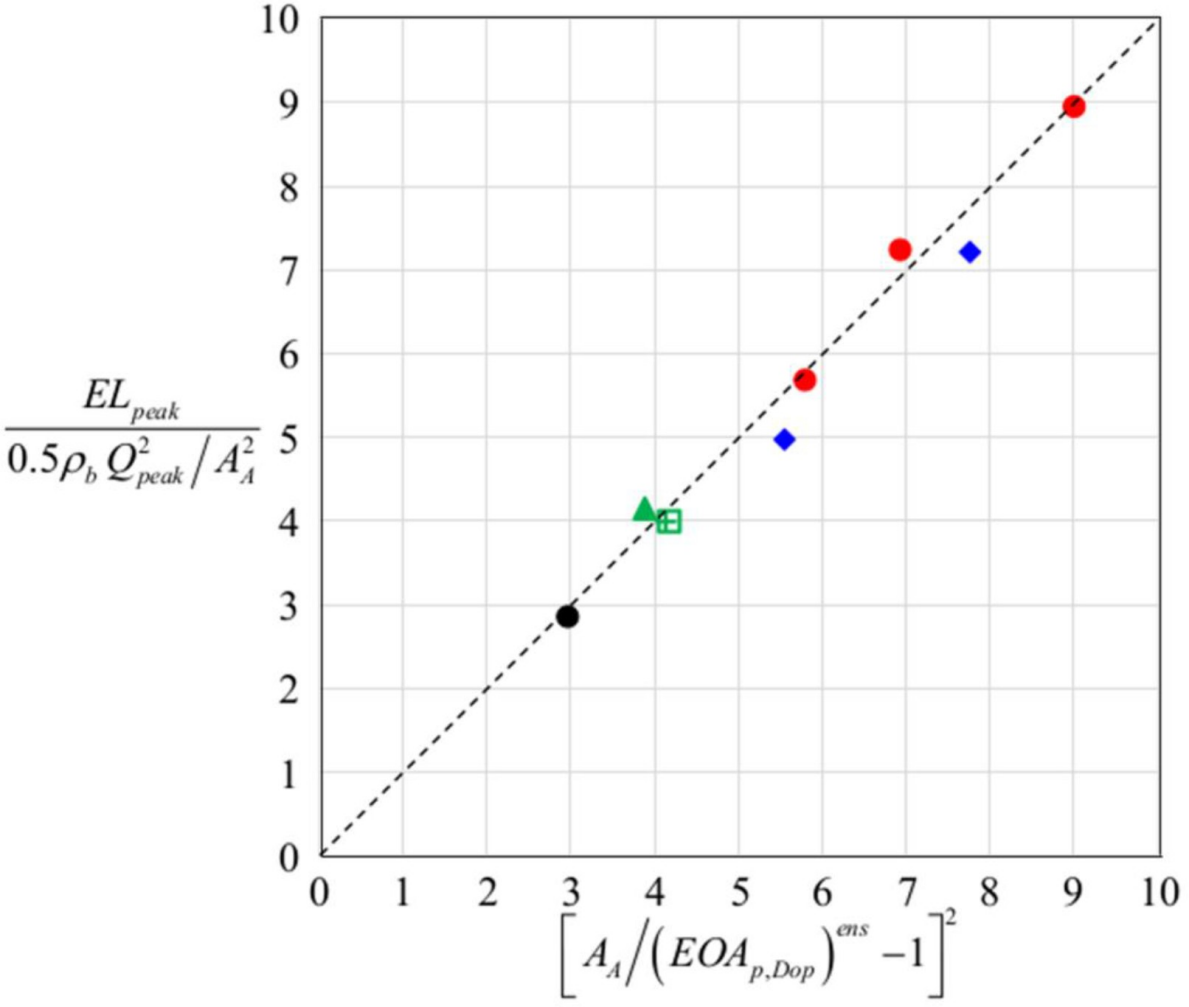
Left and right hand sides of Eq (7) for the cases considered in present study. No pannus (black circle); circular panni with *h* = 0.12*D*, 0.13*D* and 0.14*D* (red circles); upper semi-circular pannus with *h* = 0.12*D* (green triangle); right and left semi-circular panni with *h* = 0.12*D* (green square and cross symbols, respectively); and restricted opening angles of 15° and 20° (blue diamonds).

In practice, *EL*_*peak*_ can be predicted by Eq (7) with measured (*EOA*_*p*,*Dop*_)^*ens*^ [57]. Therefore, it is important to see how accurate Eq (7) is. Both sides of Eq (7) are obtained from present numerical simulation for various cases and plotted in Fig 12, which indicates that the prediction by Eq (7) is quite accurate. Table 1 provides the peak energy loss coefficient for the cases considered in this study. In the case of no pannus, *C*_*EL*_ = 2.85 but *C*_*EL*_ = 4.83–8.95 for circular panni, indicating that the circular panni increase the peak energy loss about two to three times that by BMHV without pannus. For the cases of semi-circular panni, their energy losses are smaller than but comparable to those of circular panni. The cases of opening angle restriction considered also produce significant energy losses.

Now, let us discuss some clinical observations in connection with the results of present numerical simulation. For a patient implanted with a 21-mm Advancing The Standard (ATS) open-pivot mechanical heart valve, the opening angle was restricted from 5° to 31° even in no direct contact between pannus and leaflets, and the peak maximum pressure drop obtained from the Bernoulli equation and measured velocities was increased from 20 mmHg to 57 mmHg by pannus formation [31]. In the present study, the oscillatory motions of the leaflets are prominent at the end of valve opening and the start of valve closing due to the periodic generation of vortex rings for the circular pannus with *h* = 0.12*D*. The peak maximum pressure drops are 8 mmHg and 22 mmHg for no pannus and circular pannus with *h* = 0.12*D*, respectively. Also, as the pannus height increases, the oscillatory motions of the leaflets become severer at the end of the opening phase and the period of fully open phase becomes shorter (e.g., the case of upper semi-circular pannus). A patient implanted with a 21-mm Sorin Bicarbon showed an intermittent incomplete closure of one of the two leaflets (closing angle of 60°) even in no direct contact between pannus and leaflets (normal complete closing angle is 70°), which resulted in intermittent severe aortic regurgitation [28]. In the present study, incomplete valve closing as well as asymmetric motion of the leaflets are observed during the closing phase due to the asymmetric distribution of flow structures around the leaflets by the upper semi-circular pannus with *h* ≥ 0.1*D*. Borazjani and Sotiropoulos [9] investigated the effect of implantation orientation of a BMHV in an anatomically realistic aorta on the leaflet motions and flow patterns, and observed that completion of valve closing was delayed further as the asymmetric motion of two leaflets was severer due to the asymmetric distribution of pressure induced by the curved ascending aorta.

Finally, we would like to mention limitations of our numerical approach. First, the inflow rates were kept to be same for all the cases considered since they are unknown *a priori* in the presence of pannus. The restricted opening angle and incomplete closing of the leaflets observed in the present study significantly change the pressure drop between the inlet and outlet, which may eventually change the inflow rates. One may consider an application of a reduced circulation model which may provide a more realistic inflow rate as well as the outlet pressure boundary condition. Even in this case, however, appropriate model constants should be provided *a priori* [58, 59]. Thus, to the best of our knowledge, the change in the inflow rates due to the presence of pannus is *a priori* unknown unless in vivo experiments are performed. Second, we modelled LVOT and ascending aorta as rigid straight pipes. Tullio et al. [15] conducted numerical simulations for rigid and flexible aortic roots, respectively, and showed that their leaflet motions were very similar to each other. Le and Sotiropoulos [60] conducted a numerical simulation for a BMHV implanted in anatomic left ventricle and aorta configuration with a lumped parameterization approach for reconstructing physiologic left ventricle kinematics. They showed that valve closing was slightly asymmetric due to highly three-dimensional retrograde flow coming back into the left ventricle and leaflets during the diastole phase, but incomplete valve closing was not observed. Therefore, in the presence of severe asymmetric pannus, incomplete valve closing or valve closing delay may be observed in a real left ventricle and aorta configuration. Third, pannus was considered as a non-deformable structure in the present study. Although pannus is composed of fibrous tissue [38, 61, 62] and is possibly deflected according to flow, it is quite rigid during the systole and diastole phases [24]. Therefore, the present results would be still valid even after the flexibility of pannus is considered.

## Conclusions

In the present study, we investigated the effects of the pannus shape (circular or semi-circular ring), implantation location (upper, left and right semi-circular panni) and heights (*h* = 0.07*D* ~ 0.14*D*) on the pressure drop across the pannus and BMHV. The pressure drop increased with increasing pannus height, but was not much affected by the orientation of semi-circular pannus formation. However, the flow fields were significantly changed by the orientation of asymmetric pannus formation. For upper semi-circular pannus, incomplete or delayed valve closing was observed. These abnormal behaviors were caused by asymmetric distributions of shear layer vortices behind the pannus and their interaction with the leaflets, which caused early start of valve closing for the upper leaflet. Therefore, in medical treatments, an asymmetric pannus reaching a critical height (e.g., *h* ≥ 0.1*D* in the present study) that causes abnormal motions of two leaflets may have to be removed to reduce a regurgitation volume.

Indices that diagnose the condition of BMHV patients with pannus may be suggested as the maximum pressure drop [21–32], net pressure drop, and peak energy loss [57]. We showed in this study that the maximum pressure drop is not necessarily proportional to the net pressure drop. The magnitude of peak energy loss provides overall quantitative information on the conditions of BMHV patients with and without pannus, and thus it is believed to be a good diagnostic tool. The peak energy loss was accurately predicted from the ensemble-averaged peak effective orifice area. Therefore, this parameter may be used for the design of a mechanical heart valve with high performance.
